# Gestational Risk as a Determining Factor for Cesarean Section according to the Robson Classification Groups

**DOI:** 10.1055/s-0040-1718446

**Published:** 2021-01-19

**Authors:** Karina Biaggio Soares, Vanessa Cristina Grolli Klein, José Antônio Reis Ferreira de Lima, Lucas Gadenz, Larissa Emile Paulo, Cristine Kolling Konopka

**Affiliations:** 1 Department of Gynecology and Obstetrics, Universidade Federal de Santa Maria, Santa Maria, RS, Brazil

**Keywords:** pregnancy, high-risk pregnancy, parturition, cesarean section, robson classification, gestação, gravidez de risco, trabalho de parto, cesárea, classificação de robson

## Abstract

**Objective** To analyze and compare the frequency of cesarean sections and vaginal deliveries through the Robson Classification in pregnant women attended at a tertiary hospital in two different periods.

**Methods** Cross-sectional, retrospective study of birth records, comprising 4,010 women, conducted from January 2014 to December 2015 in the only public regional referral hospital for the care of high- risk pregnancies, located in Southern Brazil.

**Results** The overall cesarean section rate reached 57.5% and the main indication was the existence of a previous uterine cesarean scar. Based on the Robson Classification, groups 5 (26.3%) and 10 (17.4%) were the most frequent ones. In 2015, there was a significant increase in the frequency of groups 1 and 3 ( *p* < 0.001), when compared with the previous year, resulting in an increase in the number of vaginal deliveries ( *p* < 0.0001) and a reduction in cesarean section rates.

**Conclusion** The Robson Classification proved to be a useful tool to identify the profile of parturients and the groups with the highest risk of cesarean sections in different periods in the same service. Thus, it allows monitoring in a dynamic way the indications and delivery routes and developing actions to reduce cesarean rates according to the characteristics of the pregnant women attended.

## Resumo

**Objetivo** Analisar e comparar a frequência de partos cesáreos e vaginais através da classificação de Robson em gestantes atendidas em um hospital terciário em dois períodos distintos.

**Métodos** Estudo transversal retrospectivo de registros de nascimento, compreendendo 4.010 mulheres, realizado de janeiro de 2014 a dezembro de 2015 no único hospital público de referência regional para atendimento de gestações de alto risco, localizado no sul do Brasil. A via de parto foi avaliada e as mulheres foram classificadas de acordo com a Classificação de Robson.

**Resultados** A taxa geral de cesariana foi de 57,5% e a principal indicação foi a existência de cicatriz uterina por cesariana prévia. Quando aplicada a Classificação de Robson, os grupos mais frequentes foram o 5 (26,3%) e o 10 (17,4%). No ano de 2015, ocorreu um aumento significativo da frequência dos grupos 1 e 3 ( *p* < 0,001), quando comparado ao ano anterior, resultando em aumento do número de partos vaginais ( *p* < 0,0001) e redução das taxas de cesariana.

**Conclusão** A Classificação de Robson mostra ser uma ferramenta útil para identificar o perfil das parturientes e os grupos com maior risco de cesariana em diferentes períodos em um mesmo serviço. Desta forma, permite monitorar de forma dinâmica as indicações e vias de parto e desenvolver ações para redução das taxas de cesariana conforme as características das gestantes atendidas.

## Introduction

Surgical interventions are necessary when labor does not have the expected physiological progression. However, nowadays, there is a remarkable global increase in cesarean section rates, as documented in different countries worldwide. 1 These procedures help reducing maternal and neonatal morbidity and mortality when they are properly indicated. Although safe, cesarean sections comprise surgery-inherent risks such as infection, bleedings, thromboembolic events, placental abnormalities (placenta previa, placental abruption, placenta accreta) in future pregnancies, chronic pain and internal adhesions. 2 In addition, there are newborn-associated risks such as prematurity, transient tachypnea or respiratory distress syndrome. 2 3

According to the World Health Organization (WHO), childbirth care aims at assuring the safety of mothers and newborns by intervening as little as possible in this process and by performing cesarean sections in case of real need. The internationally accepted ideal cesarean section rate ranges from 10 to 15% of childbirths; this range is based on lack of benefit on mortality rates when cesarean sections exceed 10% of childbirths, as shown in previous studies. 4

However, Brazilian rates are much higher than the established limit. According to Nakamura-Pereira et al., 5 cesarean sections account for 51.9% of childbirths in the country. In this study, high-risk pregnant women had significantly greater cesarean section rates compared with low-risk women in the public sector. Older primiparous and more educated pregnant women who have access to private services are more susceptible to abdominal delivery indications based on nonclinical factors. 6 The increased number of cesarean deliveries changes from region to region in the country, mainly between the public and private care sectors. Southern Brazil has one of the highest cesarean section rates in the country; it accounted for 58,1% of all childbirths in 2010, whereas the Southeastern region accounted for 58.3% and the Midwestern region for 57.4%. The Northern and Northeastern regions recorded the lowest cesarean delivery indices in 2010–44.4% and 41.8%, respectively. 7 The number of cesarean sections, which presented an upward curve, decreased by 1.5% in 2015; 55.5% of 3 million deliveries performed in Brazil were cesarean sections, whereas 44.5% were vaginal deliveries. On the other hand, if one takes into consideration only childbirths performed in the Brazilian Unified Health System (SUS, in the Portuguese acronym), the number of vaginal deliveries is higher (59.8%) than that of cesarean sections (40.2%). 8

Santa Maria County, which has one of the reference regional obstetric services for high-risk pregnancies in the countryside of the state of Rio Grande do Sul, recorded only 32.9% of vaginal deliveries in 2010. 7 Despite this finding, there are no regional studies that can be compared with national and international data.

The Robson Classification, which was developed by Robson in 2001, 9 has been suggested by the WHO as the standard instrument to evaluate and monitor cesarean section rates in different hospital services. 4 This classification allows distributing all pregnant women in groups based on individual features such as number of childbirths, number of previous cesarean sections, gestational age, fetal presentation and twin pregnancy. 9 10 Given the clarity, objectivity and easy application of this classification method, it has been used to survey, monitor and compare cesarean section rates within and between institutions; it also allows analyzing these data, as well as identifying safe alternatives to help reducing cesarean section rates. 11 12 13 14

The present study has analyzed childbirths performed at the Hospital Universitário de Santa Maria (HUSM, in the Portuguese acronym), as well as classified them based on the Robson Classification, to help better understanding the real situation of cesarean section indications and the profile of childbirths performed in the investigated institution. The results may collaborate with the creation of strategies to reduce the high cesarean rates in Brazilian institutions.

## Methods

The current research is a retrospective cross-sectional study comprising data about the hospitalization of pregnant women who had vaginal or cesarean delivery at the Obstetric Center of the HUSM (a regional reference hospital for high-risk pregnancies) from January 2014 to December 2015. Data were collected through the review of medical records. Parturients whose data did not allow their classification into Robson groups were excluded from the study.

During hospitalization for childbirth, labor care was managed according to the hospital service protocols. The evolution of births was monitored using a partogram and fetal vitality was accessed by intermittent auscultation of the fetal heart rate and cardiotocography if abnormal fetal heart rate or in high-risk pregnancies. A high-risk pregnancy was considered the one with increased risk for complications for the pregnant woman, the fetus or the newborn. Risk factors for a high-risk pregnancy were considered existing health conditions, such as high blood pressure, diabetes, thyropathies, hematopathies, infectious diseases, heart diseases, obesity, multiple births, among others.

Parturients were distributed into 10 groups based on the Robson Classification by following instructions provided in the base article. 9 Data were subjected to descriptive and analytical analyses. The chi-squared test was used to calculate differences between Robson groups in 2014 and 2015; *p* < 0.05 was set as statistically significant.

The project was approved by the Research Ethics Committee of the investigated institution (CAAE 58212416.3.0000.5346).

## Results

A total of 4,061 births were recorded in the period and 51 were excluded from the research due to incomplete data, remaining 4,010 for analysis. The mean maternal age was 26.2 (±7.1) years old, the mean number of childbirths was 1.2, the mean gestational age at birth was 37.8 weeks ( Table 1 ), and 49.4% of the pregnant women had at least one cesarean section prior to the assessed pregnancy. Of the total number of childbirths, vaginal deliveries corresponded to 1,702 (42.4%) and cesarean sections to 2,308 (57.6%) comprised cesarean sections. Sixty-one cases of fetal death (1.5%) were recorded throughout the studied period; 83.6% of them happened in preterm pregnancies.

**Table 1:** Demographic and obstetric features of Parturients

	Total ( *n* = 4,010)	Nulliparous ( *n* = 1,568)	Multiparous ( *n* = 2,442)
Maternal age	26.2 (±7.1)	21.9 (±5.78)	28.9 (±6.48)
Previous pregnancies	2.4 (±1.60)	1.1 (±0.40)	3.3 (±1.52)
Number of childbirths	1.2 (±1.44)	0 (0)	2.0 (±1.35)
Number of previous cesarean sections	0.6 (±0.98)	0 (0)	0.9 (±1.12)
Miscarriages	0.2 (±0.51)	0.1 (±0.39)	0.3 (±0.57)
Gestational age	37.8 (±4.51)	37.7 (±3.96)	37.8 (±4.83)
Hospitalization due to spontaneous labor	1,991 (49.7%)	760 (48.5%)	1,231 (50.5%)
Induced labor	1,031 (25.7%)	627 (40.0%)	404 (16.5%)
Cesarean section without labor	988 (24.6%)	181 (11.5%)	807 (33.0%)
Delivery type			
Vaginal delivery	1,693 (42.2%)	782 (49.9%)	911 (37.31%)
Instrumented delivery	9 (0.2%)	6 (0.4%)	3 (0.1%)
Cesarean section	2,308 (57.6%)	780 (49.7%)	1,528 (62.6%)

Deliveries at Hospital Universitário de Santa Maria, from January 2014 to December 2015; Mean ± standard deviation; number and percentage of cases.

Parturients were hospitalized for spontaneous delivery in 49.7% of cases, induced deliveries accounted for 25.7% of childbirths, whereas cesarean section before labor corresponded to 24.6% of cases ( Table 1 ). In the case of induced childbirths, 41.5% of nulliparous women and 57.4% of multiparous women evolved to vaginal delivery. Nulliparous women who were subjected to induced delivery evolved to cesarean section, whereas multiparous women evolved to vaginal delivery ( *p* < 0.0001).

**Fig. 1:**
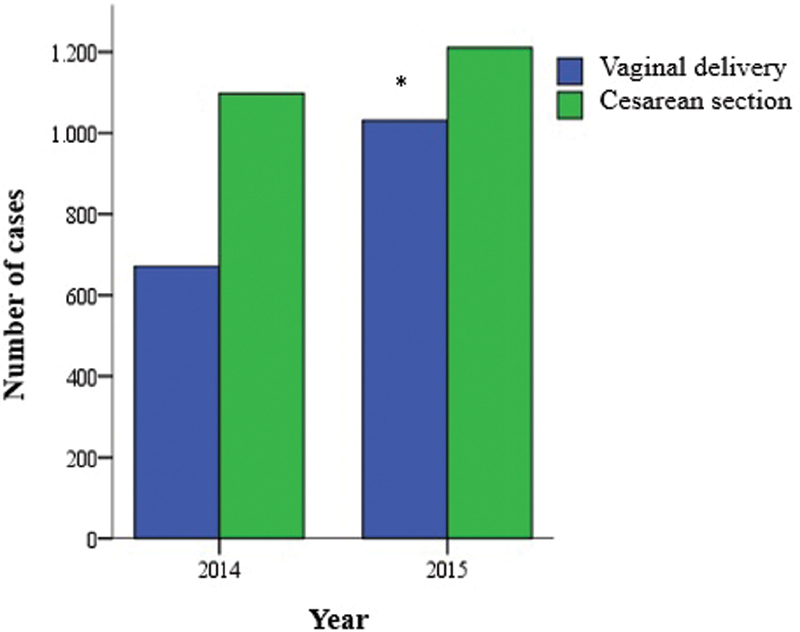
Comparison between delivery routes per year. Chi-squared test, *p < 0.0001.

Cesarean section was the most adopted delivery route (57.6% of cases); however, this rate changed depending on the number of childbirths ( Table 1 ) and on the investigated period ( Fig. 1 ); 49.7% of the total number of nulliparous women and 62.6% of the total of multiparous women evolved to cesarean section. The main indications for cesarean section comprised previous cesarean section (39.7%), nonreassuring fetal status (16.4%), cephalopelvic disproportion (12.6%), induction failure (8.4%) and pelvic presentation of the fetus (7.4%). In the studied periods (2014 and 2015), previous cesarean sections were the main indication for operative delivery and the number of previous cesareans, one or two or more did not vary ( *p* = 0.141).

**Fig. 2:**
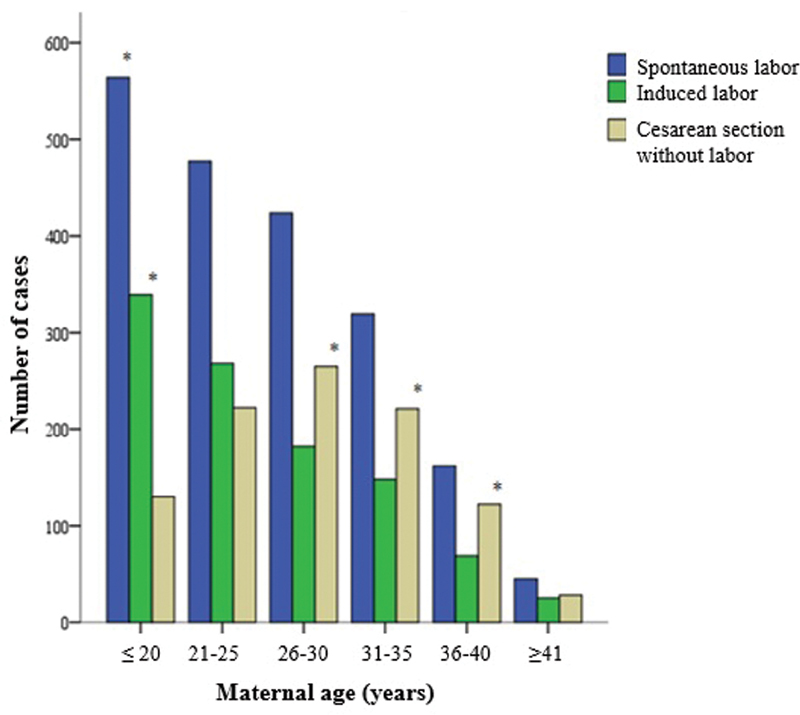
Labor onset time divided into groups, based on age group.

The stratification of pregnant women by age group revealed differences in labor onset and in delivery evolution. The age group ≤ 20 years was associated with spontaneous and induced labor onset ( *p* < 0.0001), whereas the age group between 26 and 40 years old was associated with cesarean section without labor ( *p* < 0.0001), as shown in Fig. 2 . The age group ≤ 20 years old was also associated with vaginal deliveries ( *p* < 0.0001), whereas the others were associated with cesarean sections; such association was only significant in the age group between 26 and 40 years old ( *p* < 0.0001).

Based on the comparative analysis between 2014 and 2015, the number of childbirths increased from 1,769 in 2014 to 2,241 in 2015, mainly at the expense of the total number of vaginal deliveries ( Fig. 1 ). In 2014, vaginal deliveries accounted for 37.9% of childbirths, whereas cesarean sections accounted for 62.1% of cases. In 2015, vaginal deliveries accounted for 46.1% of childbirths, whereas cesarean sections accounted for 53.9% of cases. The number of cesarean deliveries was larger in 2014 than in 2015, whereas 2015 recorded higher rates of vaginal deliveries than 2014 ( *p* < 0.0001). Another important aspect was the attendance of a greater number of healthy pregnant women in 2015. In 2014, the number of high-risk pregnancies was 17.4% and in 2015, 14.1% ( *p* = 0.016).

After data collection, parturients were distributed into 10 groups based on the Robson Classification ( Table 2 ). Most women were allocated to group 5 (26.4%), which was followed by groups 10 (17.5%) and 2 (16.0%).

**Table 2:** Cesarean section distribution based on Robson 10-group classification

Births Group	Features	2,308/4,010	Rate per group	Cesarean section rate per group	Contribution from each group
1	Nulliparous, single fetus, cephalic presentation, > 37 weeks, spontaneous labor	125/517	12.9%	24.2%	3.1%
2	Nulliparous, single fetus, cephalic presentation, > 37 weeks, induced labor or cesarean section before labor	422/642	16.0%	65.7%	10.5%
3	Multiparous, no previous cesarean section, single fetus, cephalic presentation, > 37 weeks, spontaneous labor	59/491	12.2%	12.0%	1.5%
4	Multiparous, no previous cesarean section, single fetus, cephalic presentation, > 37 weeks, induced labor or cesarean section before labor	181/364	9.1%	49.7%	4.5%
5	Multiparous with at least 1 previous cesarean section, single fetus, cephalic presentation, > 37 weeks	930/1,057	26.4%	80.0%	23.2%
6	Nulliparous, single fetus in pelvic presentation	80/81	2.0%	98.8%	2.0%
7	Multiparous, single fetus in pelvic presentation, likelihood of previous cesarean section	76/78	1.9%	97.4%	1.9%
8	Any woman with twin pregnancy; likelihood of previous cesarean section	64/68	1.7%	94.1%	1.6%
9	Any woman with oblique or transverse fetal presentation; likelihood of previous cesarean section	12/12	0.3%	100.0%	0.3%
10	Any woman with a single fetus in cephalic presentation, < 37 weeks, likelihood of previous cesarean section	359/700	17.5%	51.23%	8.9%

Deliveries at the Hospital Universitário de Santa Maria, from January 2014 to December 2015.

**Fig. 3:**
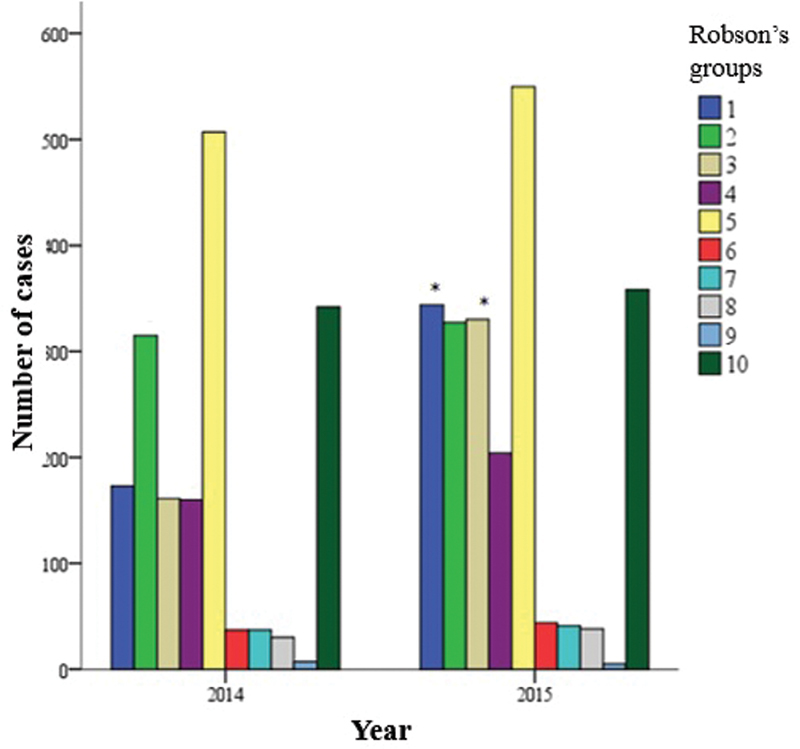
Robson groups analysis per year. Chi-squared test, *p < 0.001.

Based on the analysis of 2014 and 2015 (in separate), Robson groups 5 and 10 ( Fig. 3 ) were the most frequent ones. However, based on the association between the evaluated years (2014 and 2015) and Robson classification groups, there was a significant increase in the number of pregnant women in groups 1 and 3 in 2015 ( *p* < 0.001). The number of cases in Group 1 increased from 10.7% to 14.6% in 2014, and from 9.9% to 14.1% in Group 3. Thus, Group 1 became the third most frequent one in 2015; it took the position of Group 2 in 2014, although most women remained in groups 5 and 10.

Thus, the year of 2015 had higher number of parturients without comorbidities ( *p* = 0.016), greater number of primiparous and multiparous women with spontaneous onset of labor without previous cesarean sections ( *p* < 0.001) and higher rates of vaginal delivery ( *p* < 0.0001). However, despite the lower rate of cesarean section in 2015, there was no reduction in the need for newborns to be admitted to the neonatal therapy unit ( *p* = 0.542) nor reduction in the number of fetal deaths ( *p* = 0.777).

## Discussion

The comparison between 2014 and 2015 has shown changes in the profile of parturients; Robson groups 1 and 3 increased and, consequently, the number of vaginal deliveries also increased in 2015. This difference between the investigated years is justified by the fact that the profile of pregnant women changed between 2014 and 2015. In other words, the introduction of habitual-risk pregnant women in the group treated in the investigated service has generated a healthcare profile with tendencies similar to the ones reported by Senanayake et al. (2019), 15 who conducted a study with 7,504 women in Sri Lanka. According to the aforementioned researchers, groups 1 and 3 were the most prevalent ones, whereas Group 5 was the one that mostly increased the cesarean section rates (29.6% of indications in the aforementioned study in comparison to 23.2% in the study conducted at our institution). 15 16

Although Santa Maria County has two public maternity hospitals – one for high-risk pregnancies (HUSM) and another for habitual-risk pregnancies –, in the year 2015, the HUSM was the only reference for pregnant women treated by the SUS, both at local and regional levels, because the habitual-risk maternity hospital was temporarily closed. Thus, the HUSM conducted both high-risk and habitual-risk childbirths. This specific event has affected the total number of childbirths, as well as increased the vaginal delivery rate and the number of spontaneous childbirths in the institution in 2015, a fact that proportionally increased the number of pregnant women in groups 1 and 3. The HUSM presented results similar to the ones recorded by Tapia et al. (2016) 17 in Latin America and by Yadav et al. (2016) 18 in India when it started to perform all childbirths, not just the high-risk ones. 17 18 Therefore, the comparison between Robson Classification results and data available in the literature allowed observing that the frequency of pregnant women in each group was associated with population type, as it was also reported by Zahumensky et al. (2019), 14 who compared three Slovak centers presenting different healthcare profiles: one tertiary center with neonatal intensive care unit (NICU), intensive care unit (ICU) and two other centers that only treated pregnant women with gestational age > 32 weeks. The aforementioned study has found differences in the most recurrent Robson groups in each service, as well as cesarean section rate variations from service to service. This outcome highlighted the influence of healthcare profile on the delivery and cesarean rates in each service. 14

Based on results of the present study, most women belonged to group 5 during data collection at the HUSM – this group accounted for ∼ 25% of childbirths. This finding confirms the fact that the incidence of previous cesarean sections was the main indication for cesarean section in the investigated service. Group 10 was the second most frequent one, corresponding to births before the 37 ^th^ gestational week. This finding is justified by the fact that the investigated hospital is the only regional reference center for high-risk pregnancies, including prematurity cases.

The distribution of parturients into Robson groups changes from service to service depending on the profile of pregnant women. In comparison to other studies that have applied the Robson Classification in Brazil, a WHO survey conducted in Latin America has shown that the most frequent groups were 1 and 3. 15 According to a study conducted in a tertiary hospital in Campinas County, the main groups were 1 and 5. 11 Groups 1, 3 and 10 were the most frequent in Peru, whereas an analysis of childbirths conducted in India based on the same classification method has also found that groups 1 and 3 were the most frequent. 16 17 On the other hand, Zahumensky et al. (2019) 14 compared three different Slovak obstetric centers and found differences in the frequency of cesarean sections, mainly in groups 1, 2 and 5. Group 1, which was the largest group in the three services, accounted for the most significant difference between the absolute and relative numbers of cesarean sections. 14

Since the HUSM is a reference center for high-risk pregnancies in the central region of the state of Rio Grande do Sul and has a neonatal ICU, this hospital receives a large number of referrals for cesarean sections (indicated in the county of origin of the pregnant women), for induced or preterm labor management, as well as for other maternal and fetal complications such as premature rupture of membranes, maternal hypertension and twin pregnancy, which may lead to preterm births. These conditions make groups 2, 5 and 10 the most frequent ones in the service. Women who have had one cesarean section, as the ones in Group 5, are important determinants of the overall high cesarean section rates. Strategies focused on reducing the frequency of cesarean sections should encourage women to avoid clinically unnecessary primary cesarean sections, correctly manage labor in women with history of cesarean delivery, perform the external cephalic version for pelvic presentations, as well as vaginal delivery of twins with the first fetus in cephalic presentation, and reduce iatrogenic preterm delivery. 13 14

Another important effort focused on reducing cesarean delivery rates lies on labor induction when childbirth is indicated. According to a Portuguese study, the cesarean section rate resulting from labor induction reached 20.9%; this number corresponded to 23% of the total number of cesarean sections. According to the aforementioned study, the Foley catheter for cervical preparation was the most adopted method in labor induction cases comprising pregnant women with previous cesarean section. These cases were associated with high rates of labor induction failure and, consequently, with high rates of cesarean sections due to their direct association with Robson group 5. 18

According to studies available in the literature, induction failure rates range from 23.4 to 33.8%. 19 20 The present study recorded the following induction failure rates: 42.5% for multiparous women and 58.5% for primiparous women, which corresponded to the 4 ^th^ largest cesarean section age. 21 22 Since the HUSM is a regional reference for high-risk deliveries – including prematurity and maternal pathology cases –, the large number of induced delivery failures in this hospital has an impact on cesarean section rates. Such number also represents the risk of having another cesarean section in the future, a fact that hinders intervention measures focused on reducing cesarean section rates in the investigated service, in the short term. In addition, different induction methods and induction failure concepts may hinder the analysis of and the comparison between studies.

Despite the recommendation of the WHO to maintain cesarean section rates in, at most, 15%, the national cesarean delivery rate reached 52% in 2009; the present study recorded the following rates for the HUSM: 62.1% in 2014 and 53.9% in 2015. 7 Cesarean deliveries in Campinas County accounted for 46.6% of the total number of childbirths from 2009 to 2013. 11 Based on another WHO survey, which was conducted in several countries, cesarean sections performed in Brazil from 2010 to 2011 accounted for 47% of childbirths. 12 The cesarean section rate between 2008 and 2010 reached 30.1% in Peru, whereas in India it reached 25.1% between 2004 and 2013. 16 17 Regardless of the Human Development Index (HDI) in countries investigated by Vogel et al. (2015), 13 there is a worldwide trend toward increased numbers of obstetric interventions, as well as increased labor induction rates and larger number of cesarean sections without labor. This outcome also highlights the association between the increased number of women with previous cesarean section and the increased number of cesarean delivery indications in countries presenting moderate or low HDI. 13

According to the Department of Informatics of the SUS (DATASUS, in the Portuguese acronym), the state of Rio Grande do Sul recorded 37% of vaginal deliveries in 2014. Based on the current results, the HUSM recorded a vaginal delivery rate equal to 37.7% in 2014; this value was in compliance with the ones recorded for the state. 7 However, since the HUSM also performed habitual-risk deliveries in 2015, the vaginal delivery rate increased to 46% and reached values higher than the mean recorded for the state.

The secondary and retrospective data source used in the present study may have led to selection and measurement bias. The case loss rate was low; it accounted for 1.5% of cases, which were excluded from the analysis. It happened because the variables required to classify pregnant women based on the Robson Classification were not available in the hospital records. Despite these limitations, the main strength of the present study lies on the fact that it was the first research focused on analyzing childbirth profiles at the HUSM, which is a reference regional tertiary hospital with high-risk pregnancy representativeness in the SUS.

Although the Robson classification is a great tool to help identifying and monitoring the main groups at risk of being subjected to cesarean section, many countries and institutions have been questioning the risk of bias when the method is used to compare different maternity hospitals due to different care levels and maternal features. A recently published Italian study has shown that higher complexity hospitals are associated with higher cesarean section rates and with maternal features such as maternal age and gestational diabetes, which are seen as independent risk factors for cesarean section. 23 24

Based on the current results, the cesarean section rate in the HUSM is higher than that found in other national and international studies, but it is similar to the rate recorded for the state, as reported by Brunherotti et al. (2019), 25 who found a cesarean section rate of 60.7% in Southern Brazil. Since Group 5 presents a larger number of cesarean sections than the other groups, and since the incidence of previous cesarean section is the main indication for cesarean sections, it is necessary to take actions focused on changing the current scenario, mainly on raising the awareness about first cesarean section avoidance when it is not really necessary.

The Robson 10-group Classification System was a useful tool in the initial analysis of childbirth profiles in the service investigated herein. This classification allows monitoring the evolution of cesarean section rates in the hospital, based on actions aimed at reducing cesarean section rates, labor 26 and achieving rates closer to the ones recommended by the WHO.

## Conclusion

The Robson Classification proved to be a useful tool to identify the profile of parturients and the groups with the highest risk of cesarean sections in different periods in the same service. Thus, it allows monitoring in a dynamic way the indications and delivery routes and developing actions to reduce cesarean rates according to the characteristics of the pregnant women attended.
